# Author Correction: IFNγ-IL12 axis regulates intercellular crosstalk in metabolic dysfunction-associated steatotic liver disease

**DOI:** 10.1038/s41467-024-50607-3

**Published:** 2024-07-23

**Authors:** Randall H. Friedline, Hye Lim Noh, Sujin Suk, Mahaa Albusharif, Sezin Dagdeviren, Suchaorn Saengnipanthkul, Bukyung Kim, Allison M. Kim, Lauren H. Kim, Lauren A. Tauer, Natalie M. Baez Torres, Stephanie Choi, Bo-Yeon Kim, Suryateja D. Rao, Kaushal Kasina, Cheng Sun, Benjamin J. Toles, Chan Zhou, Zixiu Li, Vivian M. Benoit, Payal R. Patel, Doris X. T. Zheng, Kunikazu Inashima, Annika Beaverson, Xiaodi Hu, Duy A. Tran, Werner Muller, Dale L. Greiner, Alan C. Mullen, Ki Won Lee, Jason K. Kim

**Affiliations:** 1 Program in Molecular Medicine, University of Massachusetts Chan Medical School, Worcester, MA USA; 2 WCU Biomodulation Major, Department of Agricultural Biotechnology, College of Agriculture and Life Sciences, Seoul National University, Seoul, Republic of Korea; 3 Division of Nutrition, Department of Pediatrics, Faculty of Medicine, Khon Kaen University, Khon Kaen, Thailand; 4 Division of Endocrinology and Metabolism, Department of Internal Medicine, Kosin University College of Medicine, Busan, Republic of Korea; 5 Division of Endocrinology and Metabolism, Department of Internal Medicine, Soonchunhyang University Bucheon Hospital, Soonchunhyang University College of Medicine, Bucheon, Republic of Korea; 6 Division of Gastroenterology, Department of Medicine, University of Massachusetts Chan Medical School, Worcester, MA USA; 7 Division of Biostatistics and Health Services Research, Department of Population and Quantitative Health Sciences, University of Massachusetts Chan Medical School, Worcester, MA USA; 8 Division of Infection, Immunity & Respiratory Medicine, School of Biological Sciences, University of Manchester, Manchester, United Kingdom; 9 Diabetes Center of Excellence, University of Massachusetts Chan Medical School, Worcester, MA USA; 10 XO Center, Advanced Institutes of Convergence Technology, Seoul National University, Suwon, Republic of Korea; 11 Division of Endocrinology, Diabetes, and Metabolism, Department of Medicine, University of Massachusetts Chan Medical School, Worcester, MA USA

**Keywords:** Non-alcoholic fatty liver disease, Type 2 diabetes, Non-alcoholic fatty liver disease

Correction to: *Nature Communications* 10.1038/s41467-024-49633-y, published online 29 June 2024

The original version of this Article contained an error in the methods, which read “RNA-seq data analysis of TILAM in human subjects with MASLD and MASH”. The correct version now states “RNA-seq data analysis of liver genes in human subjects with MASLD and MASH”.

A second error in the methods read “We compared TILAM, including all isoforms from our prior study, and those annotated in GENCODE (v42) and COL1A1 FPKM values between MASLD and each MASH group^99^.” The correct version now states “Mapping to all annotated isoforms in GENCODE (v42) of the indicated genes (*IFNγR1, IFNγR2, IL-12A, IL-12B*, and *FGF21*) was analyzed for MASLD and each MASH group^99^.”

This has been corrected in both the PDF and HTML versions of the Article.

