# Supplementary material for: IFNγ-IL12 axis regulates intercellular crosstalk in metabolic dysfunction-associated steatotic liver disease
Source: Nat Commun. 2024 Jun 29;15:5506. doi: 10.1038/s41467-024-49633-y (PMC11217362; doi:10.1038/s41467-024-49633-y)
Supplement: Supplementary file 1 — Supplementary Information [file 41467_2024_49633_MOESM1_ESM.pdf]

Supplementary Information

IFNγ-IL12 axis regulates intercellular crosstalk in metabolic dysfunction-associated steatotic liver disease

Randall H. Friedline[1], Hye Lim Noh[1], Sujin Suk[1,2], Mahaa Albusharif[1], Sezin Dagdeviren[1], Suchaorn Saengnipanthkul[1,3], Bukyung Kim[1,4], Allison M. Kim[1], Lauren H. Kim[1], Lauren A. Tauer[1], Natalie M. Baez Torres[1], Stephanie Choi[1], Bo-Yeon Kim[1,5], Suryateja D. Rao[1], Kaushal Kasina[1], Cheng Sun[6], Benjamin J. Toles[6], Chan Zhou[7], Zixiu Li[7], Vivian M. Benoit[1], Payal R. Patel[1], Doris X.T. Zheng[1], Kunikazu Inashima[1], Annika Beaverson[1], Xiaodi Hu[1], Duy A. Tran[1], Werner Muller[8], Dale L. Greiner[1,9], Alan C. Mullen[6], Ki Won Lee[2,10], and Jason K. Kim[1,2,9,11,*]

Affiliations:

[1]Program in Molecular Medicine, University of Massachusetts Chan Medical School; Worcester, MA, USA.

[2]WCU Biomodulation Major, Department of Agricultural Biotechnology, College of Agriculture and Life Sciences, Seoul National University; Seoul, Republic of Korea.

[3]Division of Nutrition, Department of Pediatrics, Faculty of Medicine, Khon Kaen University; Khon Kaen, Thailand.

[4]Division of Endocrinology and Metabolism, Department of Internal Medicine, Kosin University College of Medicine; Busan, Republic of Korea.

[5]Division of Endocrinology and Metabolism, Department of Internal Medicine, Soonchunhyang University Bucheon Hospital, Soonchunhyang University College of Medicine; Bucheon, Republic of Korea.

[6]Division of Gastroenterology, Department of Medicine, University of Massachusetts Chan Medical School; Worcester, MA, USA.

[7]Division of Biostatistics and Health Services Research, Department of Population and Quantitative Health Sciences, University of Massachusetts Chan Medical School; Worcester, MA, USA.

[8]Division of Infection, Immunity & Respiratory Medicine, School of Biological Sciences, University of Manchester; Manchester, United Kingdom.

[9]Diabetes Center of Excellence, University of Massachusetts Chan Medical School; Worcester, MA, USA.

[10]XO Center, Advanced Institutes of Convergence Technology, Seoul National University; Suwon, Republic of Korea.

[11]Division of Endocrinology, Diabetes, and Metabolism, Department of Medicine, University of Massachusetts Chan Medical School; Worcester, MA, USA.

*Corresponding author. Email: jason.kim@umassmed.edu

Supp. Table 1.

Liver pathology evaluation for Lyz-IFNγR2$^{-/-}$ mice (KO; n=3) and WT mice (n=3) following 20 weeks on the GAN diet. Livers were freshly extracted, fixed in 10% formalin for 72 hours, and embedded in paraffin blocks. Sections (5 μm) were cut and stained with H&E and Masson's Trichrome stains. Histology slides were provided to Applied Pathology Systems (Shrewsbury, MA) for a complete pathology evaluation, including inflammation, steatosis, hepatocellular injury, and fibrosis by a board-certified pathologist. Mallory body and necrosis scores for hepatocellular injury and bile duct injury scores were 0 for both groups of mice. The total pathology scores were a sum of the portal (P), lobular (L), and perivenular (pV) scores for inflammatory infiltrate, macro-vesicular (MacV) and micro-vesicular (MicV) scores for steatosis, ballooning (Bal), acidophilic body (AB), mallory body, and necrosis scores for hepatocellular injury, bile duct injury scores, and portal, pericellular (PC), bridging (Br), and cirrhosis (C) scores for fibrosis The statistical significance of the difference in mean values was determined using a two-tailed Student's t-test.

	Inflammatory Infiltrate			Steatosis		Hepatocellular Injury		Fibrosis				Total Score
	P	L	pV	MacV	MicV	Bal	AB	P	pC	Br	C	
WT1	1	3	0	4	2	2	0	0	3	0	0	15
WT2	1	3	0	3	2	2	1	1	4	3	2	22
WT3	3	3	0	2	1	1	0	1	4	3	2	19
Avg	1.3	3.0	0.0	3.0	1.7	1.7	0.3	0.7	3.7	2.0	1.3	18.7
SE	0.3	0.0	0.0	0.6	0.3	0.3	0.3	0.3	0.3	1.0	0.7	2.0
KO1	0	2	1	3	3	0	0	0	1	0	0	10
KO2	1	2	0	4	1	1	0	0	2	0	0	11
KO3	0	3	0	4	1	1	0	0	3	0	0	12
Avg	0.3	2.3	0.3	3.7	1.7	0.7	0.0	0.0	2.0	0.0	0.0	11.0
SE	0.3	0.3	0.3	0.3	0.7	0.3	0.0	0.0	0.6	0.0	0.0	0.6
t-test	*0.10*	*0.12*	*0.37*	*0.37*	*1.00*	*0.10*	*0.37*	*0.12*	*0.07*	*0.12*	*0.12*	*0.02*

Semiquantitative score for steatosis and fibrosis: 0=no infiltrate, 1=minimal involvement (1-5%), 2=mild involvement (6-33%), 3=moderate involvement (34-66%), 4=severe involvement (>66%).

Semiquantitative score for inflammation: 0=no infiltrate, 1=minimal involvement (1 focus/HPF), 2=mild involvement (2 foci/HPF), 3=moderate involvement (3-4 foci/HPF), 4=severe involvement (>4 foci/HPF). HPF: High Power Field used to count inflammatory foci on one 200X field at the hot spot.

P: portal (periportal), L: lobular, pV: perivenular (pericentral), MacV: macro-vesicular, MicV: micro-vesicular, Bal: ballooning, AB: acidophilic body, pC: pericellular (surrounding hepatocytes), Br: bridging, C: cirrhosis.

Supp. Table 2.
qPCR primer list.

Gene	Sequence	
	**Forward primer (5' → 3')**	**Reverse primer (5' → 3')**
HPRT	TCAGTCAACGGGGGACATAAA	GGGGCTGTACTGCTTAACCAG
G6Pase	TCGGAGACTGGTTCAACCTC	AGGTGACAGGGAACTGCTTTAT
PEPCK	ACCTCCTGGAAGAACAAGGA	CTCATGGCTGCTCCTACAAA
IRS-1	GGACTTGAGCTATGACACGGG	GCCAATCAGGTTCTTTGTCTGA C
IRS-2	TGAAGGAAGCCACAGTCGTG	GTTGGTCGGAAACATGCCAA
FoxO1	TAAGGGCGACAGCAACAGCTCG	TCCGCTCTTGCCTCCCTCTGGA
Ptpn6	CCCGCTCAGGGTCACTCATA	CCCGAGTAGCGTAGTAAGGCT
STAT4	GCAGCCAACATGCCTATCCA	TGGCAGACACTTTGTGTTCCA
MAPK	CTGACCGACGACCACGTTC	CTTCGTTCACAGCTAGGTTGC
Akt	ATGAACGACGTAGCCATTGTG	TTGTAGCCAATAAAGGTGCCAT
FGF21	GTGTCAAAGCCTCTAGGTTTCTT	GGTACACATTGTAACCGTCCTC

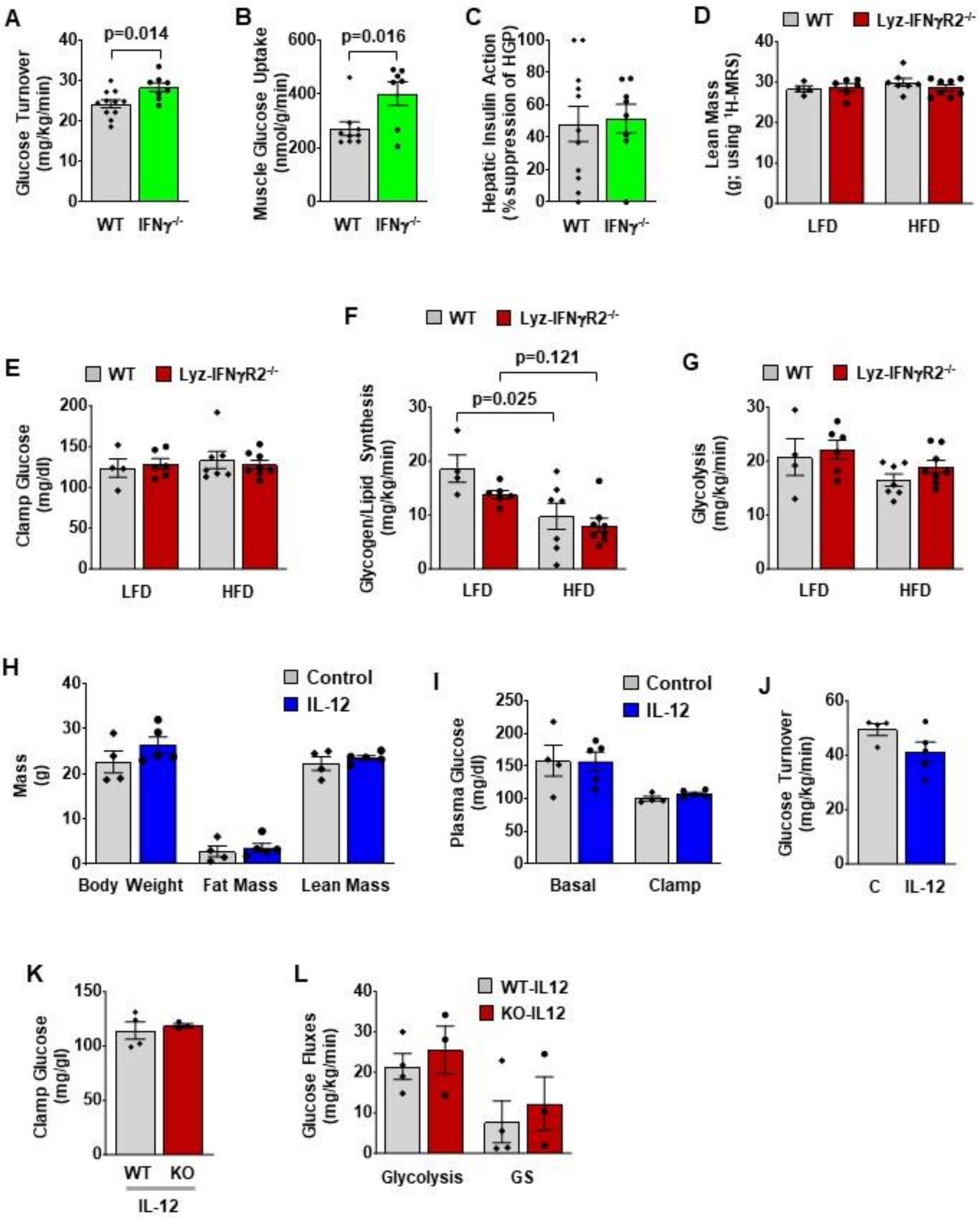

A
p=0.014
Glucose Turnover (mg/kg/min)
WT
IFNγ-/-
B
p=0.016
Muscle Glucose Uptake (nmol/g/min)
WT
IFNγ-/-
C
Hepatic Insulin Action (% suppression of HGP)
WT
IFNγ-/-
D
WT
Lyz-IFNγR2-/-
Lean Mass (g; using 1H-MRS)
LFD
HFD
E
WT
Lyz-IFNγR2-/-
Clamp Glucose (mg/dl)
LFD
HFD
F
WT
Lyz-IFNγR2-/-
p=0.121
p=0.025
Glycogen/Lipid Synthesis (mg/kg/min)
LFD
HFD
G
WT
Lyz-IFNγR2-/-
Glycolysis (mg/kg/min)
LFD
HFD
H
Control
IL-12
Mass (g)
Body Weight
Fat Mass
Lean Mass
I
Control
IL-12
Plasma Glucose (mg/dl)
Basal
Clamp
J
Glucose Turnover (mg/kg/min)
C
IL-12
K
Clamp Glucose (mg/gl)
WT
KO
IL-12
L
WT-IL12
KO-IL12
Glucose Fluxes (mg/kg/min)
Glycolysis
GS

Supp. Fig. 1. Hyperinsulinemic-euglycemic clamp in HFD-fed IFNγ$^{-/-}$ mice, Lyz-IFNγR2$^{-/-}$ mice, WT mice treated with IL-12, and HFD-fed Lyz-IFNγR2$^{-/-}$ mice with IL-12 treatment. Male IFNγ$^{-/-}$ mice (n=8) and WT mice (n=11) were fed a high-fat diet (HFD) for 6 weeks, and a standardized hyperinsulinemic-euglycemic clamp was performed to assess insulin action and glucose metabolism in awake mice. **A** Whole-body glucose turnover. **B** Insulin-stimulated glucose uptake in skeletal muscle (gastrocnemius). **C** Hepatic insulin action as insulin-mediated percent suppression of basal hepatic glucose production (HGP). Male Lyz-IFNγR2$^{-/-}$ and WT mice were fed an HFD (n=7 WT and 8 Lyz-IFNγR2$^{-/-}$ mice) or a low-fat diet (LFD; 4 WT and 6 Lyz-IFNγR2$^{-/-}$ mice) for 10 weeks prior to metabolic analyses. **D** Whole-body lean mass, measured using ^{1}H-magnetic resonance spectroscopy (MRS). **E** Plasma glucose levels during insulin clamp. **F** Whole-body glycogen plus lipid synthesis during insulin clamp. **G** Whole-body glycolysis during insulin clamp. Mouse recombinant IL-12 (0.25 µg/hour; n=5) or saline (C, Control; n=4) was intravenously infused for 4 hours in male C57BL/6J mice for hyperinsulinemic-euglycemic clamps. **H** Body weight and whole-body fat/lean mass (measured using ^{1}H-MRS). **I** Plasma glucose levels at basal state and during insulin clamp. **J** Whole-body glucose turnover. Male Lyz-IFNγR2$^{-/-}$ mice (KO; n=3) and WT mice (n=4) were fed an HFD for 12 weeks, and mouse recombinant IL-12 was chronically administered via osmotic pumps (1.2 ng/day/g body weight) during the last 2 weeks of HFD. **K** Plasma glucose levels during insulin clamp. **L** Whole-body glycolysis and glycogen/lipid synthesis (GS) during insulin clamp. Data are presented as mean ± SEM values. The statistical significance of the difference in mean values between IFNγ$^{-/-}$ mice versus WT mice, IL12-treated mice vs. Controls, and IL12-treated Lyz-IFNγR2$^{-/-}$ mice versus IL12-treated WT mice was determined using a two-tailed Student's t-test (Fig. A-C, H-J, and K-L). The statistical significance of the difference in mean values between Lyz-IFNγR2$^{-/-}$ mice and WT mice fed with an HFD or LFD was determined using a one-way analysis of variance (ANOVA) with Tukey's multiple comparison test for post-hoc analysis (Fig. D-G).

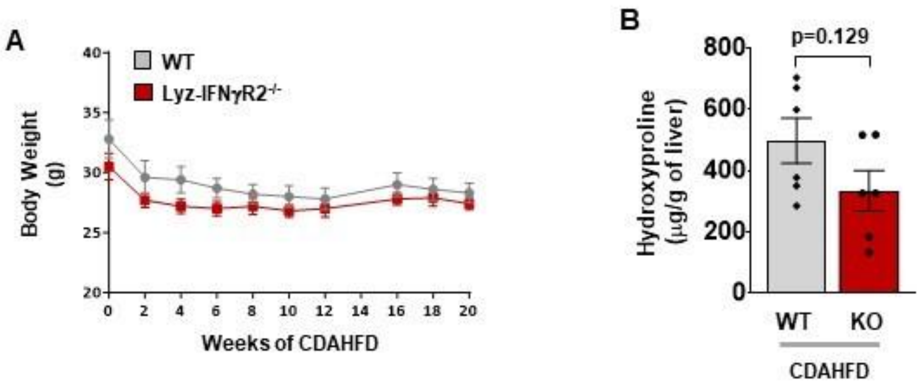

Supp. Fig. 2. Effects of CDAHFD on body weight and liver fibrosis in Lyz-IFNγR2$^{-/-}$ mice. Male Lyz-IFNγR2$^{-/-}$ and WT mice were fed a choline-deficient L-amino acid HFD (CDAHFD) for 20 weeks starting at ~8 months of age (n=6 per genotype). **A** Longitudinal changes in body weights during 20 weeks of a CDAHFD. **B** Hydroxyproline content in liver samples collected from WT and Lyz-IFNγR2$^{-/-}$ (KO) mice after 20 weeks of the GAN diet. Data are presented as mean ± SEM values. The statistical significance of the difference in mean values was determined using a two-tailed Student's t-test.
