# Supplementary material for: IFNγ-IL12 axis regulates intercellular crosstalk in metabolic dysfunction-associated steatotic liver disease
Source: Nat Commun. 2024 Jun 29;15:5506. doi: 10.1038/s41467-024-49633-y (PMC11217362; doi:10.1038/s41467-024-49633-y)
Supplement: Supplementary file 3 — Reporting Summary [file 41467_2024_49633_MOESM3_ESM.pdf]

nature portfolio

Corresponding author(s): Jason K. Kim

Last updated by author(s): Jun 4, 2024

Reporting Summary

Nature Portfolio wishes to improve the reproducibility of the work that we publish. This form provides structure for consistency and transparency in reporting. For further information on Nature Portfolio policies, see our Editorial Policies and the Editorial Policy Checklist.

Statistics

For all statistical analyses, confirm that the following items are present in the figure legend, table legend, main text, or Methods section.

n/a	Confirmed	
☐	☒	The exact sample size (n) for each experimental group/condition, given as a discrete number and unit of measurement
☐	☒	A statement on whether measurements were taken from distinct samples or whether the same sample was measured repeatedly
☐	☒	The statistical test(s) used AND whether they are one- or two-sided<br>*Only common tests should be described solely by name; describe more complex techniques in the Methods section.*
☐	☒	A description of all covariates tested
☐	☒	A description of any assumptions or corrections, such as tests of normality and adjustment for multiple comparisons
☐	☒	A full description of the statistical parameters including central tendency (e.g. means) or other basic estimates (e.g. regression coefficient) AND variation (e.g. standard deviation) or associated estimates of uncertainty (e.g. confidence intervals)
☐	☒	For null hypothesis testing, the test statistic (e.g. F, t, r) with confidence intervals, effect sizes, degrees of freedom and P value noted<br>*Give P values as exact values whenever suitable.*
☒	☐	For Bayesian analysis, information on the choice of priors and Markov chain Monte Carlo settings
☒	☐	For hierarchical and complex designs, identification of the appropriate level for tests and full reporting of outcomes
☒	☐	Estimates of effect sizes (e.g. Cohen's d, Pearson's r), indicating how they were calculated

Our web collection on statistics for biologists contains articles on many of the points above.

Software and code

Policy information about availability of computer code

Data collection	N/A
Data analysis	Microsoft Excel for Microsoft 365 MSO (Version 2312), GraphPad Prism Version 10.1.2 (324)

For manuscripts utilizing custom algorithms or software that are central to the research but not yet described in published literature, software must be made available to editors and reviewers. We strongly encourage code deposition in a community repository (e.g. GitHub). See the Nature Portfolio guidelines for submitting code & software for further information.

Data

Policy information about availability of data

All manuscripts must include a data availability statement. This statement should provide the following information, where applicable:

- Accession codes, unique identifiers, or web links for publicly available datasets
- A description of any restrictions on data availability
- For clinical datasets or third party data, please ensure that the statement adheres to our policy

The minimum datasets generated and/or analyzed during the current study that are necessary to interpret, verify and extend the research in the article are available from the corresponding author. Previously published datasets of human liver tissue were obtained from GSE135251. All scientific data and metadata generated in this study will be deposited in the National Mouse Metabolic Phenotyping Center database that will be accessible to the public upon creation of a user account.

Field-specific reporting

Please select the one below that is the best fit for your research. If you are not sure, read the appropriate sections before making your selection.

☒ Life sciences ☐ Behavioural & social sciences ☐ Ecological, evolutionary & environmental sciences

For a reference copy of the document with all sections, see nature.com/documents/nr-reporting-summary-flat.pdf

Life sciences study design

All studies must disclose on these points even when the disclosure is negative.

Sample size	The sample size for each study was calculated based on prior experimental data analysis and to detect at >20% difference between groups, assuming ~30% standard deviation, with sufficient power at >90%.
Data exclusions	There were no data exclusions.
Replication	The reproducibility of the experimental findings for in vivo metabolic studies was confirmed by multiple batches of mice showing consistent effects and different measurement parameters for each metabolic pathway showing consistent effects. The reproducibility of the experimental findings for molecular studies were confirmed by measuring protein and mRNA levels and showing consistent effects on target genes/proteins.
Randomization	Mice were randomized based on their genotypes and experimental diets (high-fat diet, MASH diets), and plasma/tissue samples were randomized for biochemical and molecular experiments.
Blinding	Experimental groups were not blinded to the researchers performing the experiments since all experimental groups were represented on each day of the experiment in order to minimize the effects of day-to-day variability of given experiments on the study.

Reporting for specific materials, systems and methods

We require information from authors about some types of materials, experimental systems and methods used in many studies. Here, indicate whether each material, system or method listed is relevant to your study. If you are not sure if a list item applies to your research, read the appropriate section before selecting a response.

Materials & experimental systems

n/a	Involved in the study	
☐	☒	Antibodies
☒	☐	Eukaryotic cell lines
☒	☐	Palaeontology and archaeology
☐	☒	Animals and other organisms
☒	☐	Human research participants
☒	☐	Clinical data
☒	☐	Dual use research of concern

Methods

n/a	Involved in the study	
☒	☐	ChIP-seq
☒	☐	Flow cytometry
☒	☐	MRI-based neuroimaging

Antibodies

Antibodies used	Cell Signaling, 3025, Insulin receptor β (4B8), 6; Millipore, 06-248, IRS-1, 2953805; Cell Signaling, 3089, IRS-2 (L1326), 3; Cell Signaling, 9272, Akt, 27; Cell Signaling, 4060, Phospho-Akt (Ser473) (D9E) XP, 23; Cell Signaling, 2880, Foxo1 (C29H4), 14; Cell Signaling, 2653, STAT4 (C46B10), 4; Cell Signaling, 5267, Phospho-Stat4 (Tyr693), 3
Validation	All antibodies were used within the approved applications and species reactivity claims by the manufacturer. "To ensure product performance, we validate all CST® antibodies, assay kits, and reagents to ensure optimal performance in the approved applications shown on our product web pages." (Cell Signaling) "Our scientists are constantly experimenting with design and development to produce the most precise, dependable antibodies in the world. Our methodology and validation yield reliable evidence that allows us to repeatedly exceed our standards. That is why we are able to produce high quality antibodies that work. Our efforts, our battles, give you the immunity you need to succeed. Our failures, converted to success, make you Anti-Fail." (Millipore)<br>3025, UniProt ID: P06213, Entrez-Gene Id: 3643<br>06-248, UniProt ID: P35568, NCBI Accession: NP_005535,<br>3089, UniProt ID: Q9Y4H2, Entrez-Gene Id: 8660<br>9272, UniProt ID: P31751 , Q9Y243 , P31749, Entrez-Gene Id: 208 , 10000 , 207<br>4060, UniProt ID: P31751 , Q9Y243 , P31749, Entrez-Gene Id: 208 , 10000 , 207<br>2880, UniProt ID: Q12778, Entrez-Gene Id: 2308<br>2653, UniProt ID: Q14765, Entrez-Gene Id: 6775<br>5267, UniProt ID: Q14765 (validation of antibody-target), Entrez-Gene Id: 6775 (validation of mouse gene ortholog)

Animals and other organisms

Policy information about studies involving animals; ARRIVE guidelines recommended for reporting animal research

Laboratory animals	We used C57BL/6J mice as wild-type mice for IL-12 treatment experiments. We developed a new transgenic mouse model with a conditional loss of IFNgamma signaling in myeloid cells (Lyz-IFNgR2 KO mice). Lyz-IFNgR2 KO mice were generated by cross-breeding IFNgR2-floxed mice with LysM-Cre mice on C57BL/6J background. The current study examined male mice because female C57BL/6J mice are resistant to diet-induced obesity and insulin resistance, a primary endpoint of current study. In order to study female mice, we would need to perform a ovariectomy procedure for the female C57BL/6J mice to become obese, and such procedure creates other variables (e.g., lacking ovarian hormones) that may confound data interpretation. However, we understand the importance of studying both sexes, and future experiments will examine female mice.
Wild animals	N/A
Field-collected samples	N/A
Ethics oversight	Institutional Animal Care and Use Committee of the University of Massachusetts Chan Medical School (protocol number #202000104)

Note that full information on the approval of the study protocol must also be provided in the manuscript.
